# Translation, Validity, and Reliability of the Minnesota Tobacco Withdrawal Scale (MTWS) in Indonesian

**DOI:** 10.7759/cureus.87986

**Published:** 2025-07-15

**Authors:** Tribowo Ginting, Martina W Nasrun, Kristiana Siste, Jacub Pandelaki, Aria Kekalih, Melva Louisa, Agus Dwi A Susanto, Diah S Utami, Wresti Indriatmi, Immanuel N Tarigan, Ricky Nathaniel, Alya R Trishna

**Affiliations:** 1 Psychiatry, Universitas Indonesia, Persahabatan Hospital, Jakarta, IDN; 2 Psychiatry, Universitas Indonesia, Dr. Cipto Mangunkusumo National Central General Hospital, Jakarta, IDN; 3 Radiology, Universitas Indonesia, Dr. Cipto Mangunkusumo National Central General Hospital, Jakarta, IDN; 4 Community Medicine, Universitas Indonesia, Jakarta, IDN; 5 Pharmacology and Therapeutics, Universitas Indonesia, Jakarta, IDN; 6 Pulmonology and Respiratory Medicine, Faculty of Medicine Universitas Indonesia - Persahabatan Hospital, Jakarta, IDN; 7 Psychiatry, Indonesia National Narcotics Board, Jakarta, IDN; 8 Dermatology, Universitas Indonesia, Dr. Cipto Mangunkusumo National Central General Hospital, Jakarta, IDN; 9 Insurance Claim Management, Dr. Cipto Mangunkusumo National Central General Hospital, Jakarta, IDN; 10 Psychiatry, Persahabatan Hospital, Jakarta, IDN

**Keywords:** mtws questionnaire, reliability, smoking cessation, translation, validity

## Abstract

Introduction

Tobacco addiction remains a major public health issue in Indonesia, with smoking cessation often hindered by withdrawal symptoms. The Minnesota Tobacco Withdrawal Scale (MTWS) is a widely used assessment tool, but no validated Indonesian version has been available. This study aimed to translate, validate, and assess the reliability of the Indonesian MTWS.

Methods

A cross-sectional study was conducted from August to December 2022 with 174 adult smokers. The MTWS was translated and reviewed by five addiction specialists. Content validity was assessed using the Scale-Level Content Validity Index (S-CVI), construct validity through correlation analysis and exploratory factor analysis (EFA), and reliability via Cronbach's alpha.

Results

The content validity analysis showed that six statements had an Item Content Validity Index (I-CVI) score of 1.0, while 11 statements scored 0.8, resulting in an overall Scale Content Validity Index/Average (S-CVI/Ave) score of 0.87, indicating strong content validity. Construct validity testing revealed that all items had correlation coefficients exceeding the critical r-value (0.124), confirming their validity. EFA indicated a strong factor structure, with a Kaiser-Meyer-Olkin (KMO) value of 0.970 and a Bartlett's Test of Sphericity p-value <0.001. Reliability testing demonstrated high internal consistency, with a Cronbach's alpha of 0.923.

Conclusion

The Indonesian MTWS is a valid and reliable tool for assessing smoking withdrawal symptoms, supporting healthcare professionals in smoking cessation efforts.

## Introduction

Tobacco addiction is still a major health problem in Indonesia. From the Global Adult Tobacco Survey 2021 held by the Indonesian Ministry of Health, the numbers of adult smokers grew by as much as 8.8 million people in a span of ten years (from 2011 to 2021), as in 2018, 33.8% of both sexes adult populations in Indonesia are smokers ​[1,2]. Its catastrophic effect not only impacts the health burden-smoking is a major factor of cerebrovascular attack, ischemic heart disease, and chronic obstructive pulmonary disease (COPD), which mortality rate increases by 29.2%, 29.0%, and 10.5% respectively from 2007 to 2017, but also the economic burden in Indonesia ​[3]. The economic cost of smoking for the entire population of Indonesia in 2019 ranged from Rp 184.36 trillion to Rp 410.76 trillion, as stated by Meilissa et al. (2021), which resulted in 61.2% to 91.8% of the 2019 deficit of Indonesia's National Health Insurance System (Badan Penyelenggara Jaminan Sosial Kesehatan (BPJS))​ [4]. 

Given the profound health and economic impacts of tobacco use in Indonesia, addressing smoking cessation is critical, not only to reduce the national burden but also to support individuals in managing the challenges of quitting, particularly the often debilitating symptoms of tobacco withdrawal. In the process of smoking cessation, the symptoms of tobacco withdrawal may vary from person to person. The DSM-5 lists major symptoms of tobacco withdrawal-including irritability, frustration, anger, depressed mood, anxiety, difficulty in concentrating, increased appetite, and restlessness; these symptoms occur when blood or tissue concentration of nicotine decline in patients with chronic use of tobacco and often interrupt patients' daily functions, quality of life, and may cause significant distress both socially and occupationally ​[5]. 

To assess tobacco withdrawal symptoms, healthcare professionals may use the Minnesota Tobacco Withdrawal Scale (MTWS). It is a self-reported questionnaire based on what symptoms the patients are experiencing in the past 24 hours. It consists of major eight items of questions based on DSM-5 symptoms: 1) desire to smoke, 2) anger, irritability, or frustration, 3) anxiety or nervousness, 4) difficulty in concentrating, 5) impatience or restlessness, 6) hunger, 7) insomnia or awakening at night, and 8) depressive mood. There are also other possible symptoms questioned, such as constipation, dizziness, drowsiness, coughing, and several more. Items are rated from a scale of 0 to 4 (0: none, 1: slight, 2: mild, 3: moderate, 4: severe). These items are then added up, and it in total ranged from 0 to 32 ​[6]. 

In the movement of smoking cessation, monitoring patients' progress is an essential step, which can be done by using MTWS. MTWS is available in various languages, but it has yet to be translated into Indonesian. This study aimed to develop an Indonesian version of the MTWS and evaluate its content validity, construct validity, and internal reliability. With this Indonesian translation of the Minnesota Tobacco Withdrawal Scale, we hope to push the national smoking cessation movement forward.

## Materials and methods

Research design 

This research utilized a cross-sectional design and was carried out between August and December 2022, collecting data through an online questionnaire distributed via Google Forms. Ethical approval for the study was obtained from the Research Ethics Committee of the Faculty of Medicine, University of Indonesia (NO: KET-727/UN2.F1/ETIK/PPM.00.02/2022). 

Subjects 

The participants in this study were adult smokers residing in Indonesia. A sample size of 174 was determined based on the rule of thumb. Participants were recruited through consecutive sampling, through online and offline flyers distributed in Persahabatan Hospital, East Jakarta. Eligibility criteria included adults aged 18 and older who had been actively smoking for at least six months and exclusively used tobacco cigarettes. Individuals with medical or psychiatric conditions that impaired their ability to communicate or complete the questionnaire were excluded from the study. 

Translation 

The validation process of the MTWS questionnaire was not preceded by permission from the questionnaire maker because this questionnaire is an open questionnaire that is free to be translated. Each item of the MTWS questionnaire was translated by two sworn translators to further discuss the translation results that would be used, and then back-translated from Indonesian to English to test consistency. 

Validity testing 

Content Validity 

Content validation was performed through a panel discussion, utilizing the Scale-Level Content Validity Index (S-CVI). Experts evaluated each question using a four-point scale, where 1 represented "not relevant" and 4 represented "relevant." Scores of 1-2 were classified as not relevant, while scores of 3-4 were deemed relevant. The Scale Content Validity Index/Average (S-CVI/Ave) was calculated by dividing the total of I-CVI scores by the number of items assessed. An S-CVI/Ave value exceeding 0.9 was considered indicative of strong content validity ​[7]. 

Construct Validity 

Construct validity was evaluated by analyzing the correlation between each individual items and the overall questionnaire. The test compared the correlation coefficients of the items with a predetermined critical value. Items were deemed valid if their correlation coefficient exceeded the critical value. The assessment was performed using SPSS Statistics version 25 (IBM Inc., Armonk, New York). 

Reliability Testing 

The questionnaire’s reliability was assessed by calculating Cronbach's alpha (α) to evaluate internal consistency. A Cronbach's alpha value exceeding 0.6 was considered satisfactory. The data analysis was conducted using SPSS Statistics version 25. 

## Results

Demographics

A total of 174 subjects took part in the study. The majority of participants are male (90.2%), with a mean age of 36.64 years. Most participants have at least a high school education (85%) and reside in Jakarta or Java (86.8%). The predominant type of cigarette used is filtered cigarettes (75.3%). The median smoking duration is 13.5 years, with a median daily consumption of 12 cigarettes. While 86.2% have attempted to quit smoking, only 7.5% have sought professional help. The demographic characteristics of the subjects are shown in Table 1.

**Table 1:** Demographic characteristics of subjects

Demographic characteristics		N (%)
Age (mean ± standard deviation) in years		36.64 ± 10.33
Sex	Male	157 (90.2%)
	Female	17 (9.8%)
Latest education	Junior high school	6 (3.4%)
	Senior high school	78 (44.8%)
	Bachelor’s degree	70 (40.2%)
	Masters degree	20 (11.5%)
Location	Jakarta	71 (40.8%)
	Java (outside of Jakarta)	80 (46.0%)
	Sumatera	14 (8.0%)
	Bali-Nusa Tenggara	2 (1.1%)
	Kalimantan	6 (3.4%)
	Sulawesi	1 (0.6%)
Type of cigarettes consumed	Cigarettes with a filter	131 (75.3%)
	Cigarettes without filter (kretek)	23 (13.2%)
	Both	20 (11.5%)
Duration of smoking (median (minimum-maximum)) in years		13,5 (1-50)
Daily cigarette consumption (median (minimum-maximum)) in years		12 (1-40)
Attempt to quit smoking	Yes	150 (86.2%)
	No	24 (13.8%)
Professional attempt to quit smoking	Yes	13 (7.5%)
	No	161 (92.5%)

The two translated results were discussed with experts, consisting of five doctors specialized in addiction cases. The expert discussion produced a questionnaire that was mutually agreed upon for testing. A trial was conducted on 10 subjects, and then discussed with the experts. In the trial process with 10 subjects, all subjects clearly understood the instructions, and no further input was given by the subjects. A second expert discussion was conducted. In the discussion, a comparison was also made between the original questionnaire and the back-translated questionnaire. The comparison of the English and Indonesian versions of the questionnaire is shown in Table 2.

**Table 2:** Comparison of original MTWS questionnaire and Indonesian version MTWS - Minnesota Tobacco Withdrawal Scale

Statements	English version	Indonesian version
1	Angry, irritable, frustrated	Marah, mudah tersinggung, frustrasi
2	Anxious, nervous	Cemas, gugup
3	Depressed mood, sad	Suasana perasaan depresi, sedih
4	Difficulty concentrating	Sulit berkonsentrasi
5	Increased appetite, hungry, weight gain	Nafsu makan meningkat, lapar, peningkatan berat badan
6	Insomnia, sleep problems, awakening at night	Sulit tidur, tidur bermasalah, terbangun di malam hari
7	Restless	Gelisah
8	Impatient	Tidak sabar
9	Cheerful/elated	Ceria/sangat gembira
10	Constipation	Sulit buang air besar
11	Coughing	Batuk
12	Craving to smoke	Dorongan yang kuat untuk merokok
13	Decreased pleasure from events	Berkurangnya kesenangan dalam berbagai kegiatan
14	Dizziness	Pusing
15	Drowsy	Mengantuk
16	Impulsive	Impulsif
17	Mouth ulcers	Sariawan

Validity testing

Content Validity

A panel of five experts conducted an assessment, evaluating the questionnaire's content validity. Among the 17 statements assessed, six received unanimous agreement on relevance, resulting in an Item Content Validity Index (I-CVI) score of 1.0. Meanwhile, the remaining 11 statements achieved an I-CVI score of 0.8. The questionnaire's overall Scale Content Validity Index/Average (S-CVI/Ave) was calculated at 0.87, indicating that it meets the criteria for content validity (Table 3).

**Table 3:** Content validity testing result of MTWS MTWS - Minnesota Tobacco Withdrawal Scale; I-CVI - Item Content Validity Index; S-CVI/Ave - Scale Content Validity Index/Average

Statements	Relevance
1	0.8
2	1
3	1
4	1
5	0.8
6	1
7	1
8	0.8
9	0.8
10	0.8
11	0.8
12	1
13	0.8
14	0.8
15	0.8
16	0.8
17	0.8
Total I-CVI	14.8
S-CVI/Ave	0.87

Construct Validity 

Among the 17 statement items in the MTWS questionnaire, six exhibit a strong correlation (r>0.7), seven demonstrate a moderate correlation (r=0.5-0.6), and four have a weak correlation (Table 4). However, all statement items show a calculated r-value exceeding the critical threshold (r=0.124), confirming their validity. For the four statements with weaker correlations, the expert panel reached a consensus that these items remain valid due to their clinical significance. These four statements are statement five (r=0.489), nine (r=0.431), 10 (r=0.403), and 17 (r=0.361). Consequently, they have been retained in the Indonesian version of the MTWS questionnaire.

**Table 4:** Construct validity result of the MTWS questionnaire MTWS - Minnesota Tobacco Withdrawal Scale

Statements	Calculated r value	Critical r value	Conclusion
1	0.718	0.124	Valid
2	0.675	0.124	Valid
3	0.716	0.124	Valid
4	0.726	0.124	Valid
5	0.489	0.124	Valid
6	0.681	0.124	Valid
7	0.739	0.124	Valid
8	0.739	0.124	Valid
9	0.431	0.124	Valid
10	0.403	0.124	Valid
11	0.605	0.124	Valid
12	0.667	0.124	Valid
13	0.689	0.124	Valid
14	0.682	0.124	Valid
15	0.628	0.124	Valid
16	0.706	0.124	Valid
17	0.361	0.124	Valid

**Figure 1:**
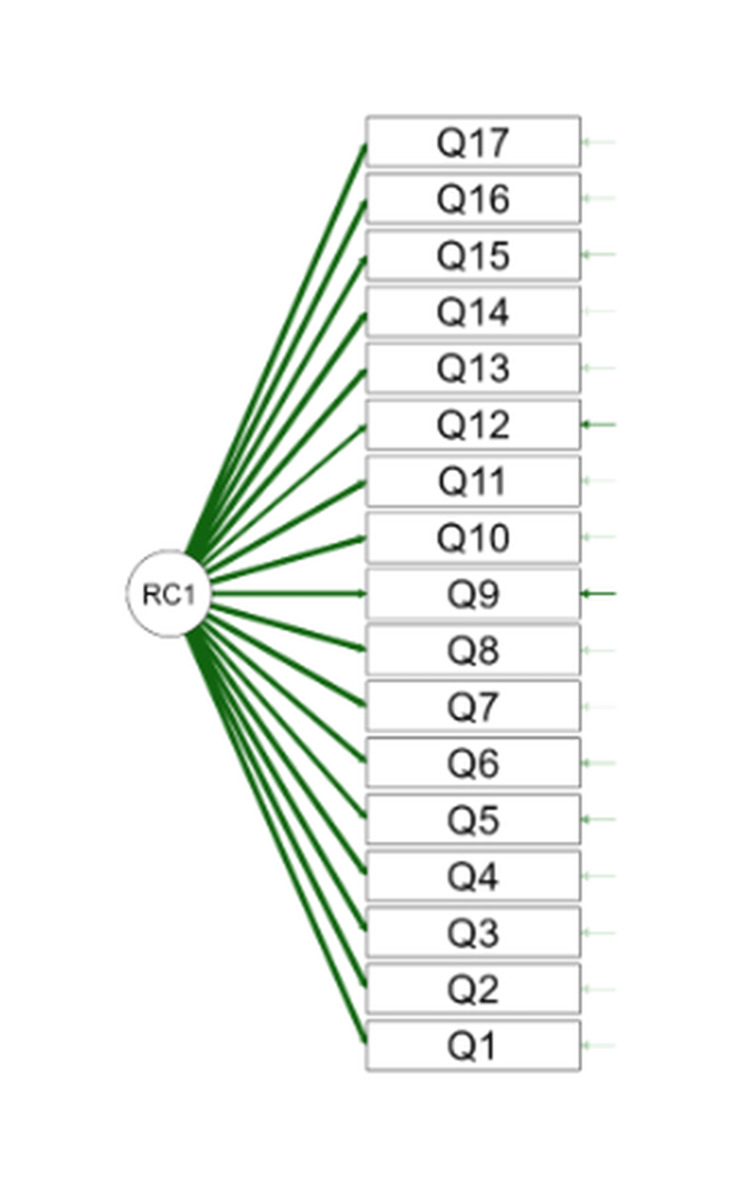
Path diagram of the Indonesian MTWS

In addition, an exploratory factor analysis (EFA) test was also conducted to assess construct validity. The Kaiser-Meyer-Olkin (KMO) value in this questionnaire was 0.970 (Table 5) with a Bartlett's Test of sphericity (BTS) value of p<0.001 (Table 6). This indicates that the number of samples and the correlation between items in this questionnaire are strong. The EFA test on all MTWS statements showed good loading factors (≥ 0.4) and only formed one domain (Table 7-9). The domain formed has an eigenvalue of more than one (14,044) and can cumulatively explain 81.6% of the variance. This questionnaire has a good goodness of fit value with a CFI value of 0.951; SRMR: 0.023, and RMSEA: 0.105 (90% CI: 0.092-0.118) (Table 10). Figure 1 shows the path diagram of Indonesian MTWS.

**Table 5:** Kaiser-Meyer Olkin test of the Indonesian MTWS MTWS - Minnesota Tobacco Withdrawal Scale; MSA - measure of sampling adequacy

Kaiser-Meyer Olkin test	MSA
Overall MSA	0.970
Q1	0.977
Q2	0.959
Q3	0.956
Q4	0.982
Q5	0.969
Q6	0.970
Q7	0.965
Q8	0.984
Q9	0.950
Q10	0.982
Q11	0.976
Q12	0.968
Q13	0.966
Q14	0.953
Q15	0.977
Q16	0.972
Q17	0.982

**Table 6:** Bartlett's test of the Indonesian MTWS MTWS - Minnesota Tobacco Withdrawal Scale

Bartlett's test		
Χ²	df	p-value
4781.399	136.000	<0.001

**Table 7:** Factor loadings of indonesian MTWS MTWS - Minnesota Tobacco Withdrawal Scale The applied rotation method is Promax.

	Factor 1	Uniqueness
Q14	0.970	0.059
Q7	0.950	0.098
Q2	0.943	0.111
Q13	0.938	0.120
Q8	0.937	0.121
Q4	0.933	0.129
Q3	0.931	0.133
Q11	0.928	0.139
Q17	0.927	0.141
Q16	0.926	0.142
Q10	0.924	0.146
Q1	0.915	0.162
Q15	0.883	0.221
Q6	0.877	0.231
Q5	0.842	0.290
Q12	0.755	0.431
Q9	0.741	0.451

**Table 8:** Factor characteristics of the Indonesian MTWS MTWS - Minnesota Tobacco Withdrawal Scale

		Unrotated solution			Rotated solution		
	Eigenvalues	SumSq. Loadings	Proportionvar.	Cumulative	SumSq. Loadings	Proportionvar.	Cumulative
Factor 1	14.044	13.874	0.816	0.816	13.874	0.816	0.816

**Table 9:** Factor Correlations of Indonesian MTWS

Factor Correlations	
	Factor 1
Factor 1	1.000

**Table 10:** Additional fit indices of the Indonesian MTWS MTWS - Minnesota Tobacco Withdrawal Scale; RMSEA - root mean square error of approximation; SRMR - standardized root mean square residual; TLI - Tucker–Lewis index; CFI - comparative fit index; BIC - Bayesian information criterion

Additional fit indices					
RMSEA	RMSEA 90% confidence	SRMR	TLI	CFI	BIC
0.105	0.092 - 0.118	0.023	0.944	0.951	-267.348

Reliability testing

The reliability test on the MTWS questionnaire showed good results with a Cronbach's alpha (α) value of 0.923. In addition, the reliability test also found that all statements on the MTWS questionnaire had a Cronbach's alpha value above 0.9.

**Table 11:** Reliability testing result of the MTWS questionnaire Indonesian version MTWS - Minnesota Tobacco Withdrawal Scale

Statements	Cronbach’s alpha	Conclusion
1	0.917	Reliable
2	0.917	Reliable
3	0.917	Reliable
4	0.916	Reliable
5	0.924	Reliable
6	0.919	Reliable
7	0.915	Reliable
8	0.915	Reliable
9	0.927	Reliable
10	0.923	Reliable
11	0.920	Reliable
12	0.921	Reliable
13	0.917	Reliable
14	0.916	Reliable
15	0.918	Reliable
16	0.916	Reliable
17	0.923	Reliable

## Discussion

This study aimed to assess the validity and reliability of the Indonesian-translated version of the MTWS questionnaire. A validated Indonesian version of the Minnesota Tobacco Withdrawal Scale (MTWS) has not been available prior to this study. Despite the widespread use of MTWS in various languages, no officially translated and tested version existed in Indonesia, highlighting the need for a culturally adapted and linguistically accurate tool to assess smoking withdrawal symptoms among Indonesian-speaking populations. With the majority of male participants (90.2%), this study's demographic characteristic is aligned with 2021's Indonesian Global Adult Tobacco Survey (GATS), which reported that 65.5% of males and 3.3% of females currently used tobacco, showing a gap for both sexes on tobacco consumption [1].

The validity and reliability assessment of the Indonesian version of the MTWS questionnaire, conducted by five experts, demonstrated strong content validity. Six statements received an Item Content Validity Index (I-CVI) score of 1.0, while 11 statements obtained a score of 0.8. Since all I-CVI values exceeded 0.79, and the final Scale Content Validity Index/Average (S-CVI/Ave) score was 0.87 (>0.8), the questionnaire was deemed content-valid. 

In terms of construct validity, the Indonesian version of the MTWS was also validated, as each statement achieved an r-value above the critical threshold (r=0.124). Furthermore, the reliability analysis showed strong internal consistency, with a Cronbach's alpha (α) of 0.923, indicating high reliability. 

The MTWS questionnaire is useful for assessing withdrawal symptoms in smokers, which is an essential step in smoking cessation ​[8]. The nicotine withdrawal syndrome is considered one of the most crucial factors for the high relapse rate in individuals undergoing smoking cessation therapy, as in Indonesia, only 15.7% of smokers have been reported to successfully quit smoking ​[9,10]. 

The MTWS questionnaire is freely accessible and available in multiple languages; however, no official Indonesian translation existed before this study. The Indonesian version can now be compared with previously published language versions. Notably, in terms of reliability, the Indonesian version exhibits a higher Cronbach's alpha (α) of 0.923 compared to the Malaysian version (0.91), the Italian version (0.84-0.87), and the Korean version (0.88) ​[11-13].

The Indonesian translation of the MTWS holds significant potential for healthcare professionals as a standardized tool to evaluate the severity of smoking withdrawal symptoms in patients. By systematically assessing withdrawal levels, healthcare workers can gain valuable insights into the challenges individuals face during the smoking cessation process. This, in turn, can inform the development of more effective intervention strategies and personalized support plans to improve cessation outcomes. Limitations of this study include gender-bias of the subjects (90.2% male vs. 9.8% female) and the subject's centered demographic area in Jakarta or Java (86.8%) which may limit generalizability, its data collection procedure through Google Forms (online) and using consecutive sampling; which may introduce self-selection and self-report bias, not assessing temporal stability of the instrument, and not including the comparison of the Indonesian MTWS with other validated instruments. As the findings support cross-sectional validity and internal consistency only, additions of test-retest reliability testing and predictive validity are encouraged for future longitudinal works.

## Conclusions

Monitoring withdrawal symptoms is particularly important, as these symptoms, such as cravings, irritability, anxiety, and restlessness, can create substantial barriers to quitting. By utilizing the MTWS, healthcare providers can better understand the specific difficulties experienced by patients, allowing them to offer timely and targeted assistance. Ultimately, this tool may contribute to advancing smoking cessation efforts in Indonesia, supporting national public health initiatives aimed at reducing tobacco dependence and promoting a healthier population. The study concluded that the Indonesian version of the MTWS questionnaire is a valid and reliable assessment tool, making it well-suited for routine use in clinical and research settings. 

## Appendices

Skala Penilaian Perilaku  

Penilaian Mandiri  

Mohon menilai diri Anda sendiri dalam periode _________ terakhir 

0 = tidak ada, 1 = sedikit, 2 = ringan, 3 = sedang, 4 = berat  

**Table 12:** MTWS English Version vs. Indonesian Version MTWS questionnaire was acquired from http://www.med.uvm.edu/behaviorandhealth/research/minnesota-tobacco-withdrawal-scale. Permission from the author was not required to translate the questionnaire as the questionnaire is available publicly for translation. MTWS - Minnesota Tobacco Withdrawal Scale

English Version		Indonesian Version	Skor (Score)				
DSM-5 Symptoms		Gejala DSM-5					
1	Angry, irritable, frustrated	Marah, mudah tersinggung, frustrasi	0	1	2	3	4
2	Anxious, Nervous	Cemas, gugup	0	1	2	3	4
3	Depressed mood, sad	Suasana perasaan depresi, sedih	0	1	2	3	4
4	Difficulty concentrating	Sulit berkonsentrasi	0	1	2	3	4
5	Increased appetite, hungry, weight gain	Nafsu makan meningkat, lapar, peningkatan berat badan	0	1	2	3	4
6	Insomnia, sleep problems, awakening at night	Sulit tidur, tidur bermasalah, terbangun di malam hari	0	1	2	3	4
7	Restless	Gelisah	0	1	2	3	4
8	Impatient	Tidak sabar	0	1	2	3	4
Other possible symptoms		Gejala lain yang mungkin	Skor (Score)				
9	Cheerful/elated	Ceria/sangat gembira	0	1	2	3	4
10	Constipation	Sulit buang air besar	0	1	2	3	4
11	Coughing	Batuk	0	1	2	3	4
12	Craving to smoke	Dorongan yang kuat untuk merokok	0	1	2	3	4
13	Decreased pleasure from events	Berkurangnya kesenangan dalam berbagai kegiatan	0	1	2	3	4
14	Dizziness	Pusing	0	1	2	3	4
15	Drowsy	Mengantuk	0	1	2	3	4
16	Impulsive	Impulsif	0	1	2	3	4
17	Mouth ulcers	Sariawan	0	1	2	3	4

Laju nadi  ___________kali per menit  

Berat badan_______________ kg 
